# Experimental environment improves the reliability of short-latency afferent inhibition

**DOI:** 10.1371/journal.pone.0281867

**Published:** 2023-02-22

**Authors:** Karishma R. Ramdeo, Ravjot S. Rehsi, Stevie D. Foglia, Claudia V. Turco, Stephen L. Toepp, Aimee J. Nelson

**Affiliations:** 1 Department of Kinesiology, McMaster University, Hamilton, Canada; 2 School of Biomedical Engineering, McMaster University, Hamilton, Canada; 3 Faculty of Medicine and Dentistry, University of Alberta, Edmonton, Canada; Universita degli Studi di Torino, ITALY

## Abstract

Evidence indicates attention can alter afferent inhibition, a Transcranial Magnetic Stimulation (TMS) evoked measure of cortical inhibition following somatosensory input. When peripheral nerve stimulation is delivered prior to TMS, a phenomenon known as afferent inhibition occurs. The latency between the peripheral nerve stimulation dictates the subtype of afferent inhibition evoked, either short latency afferent inhibition (SAI) or long latency afferent inhibition (LAI). While afferent inhibition is emerging as a valuable tool for clinical assessment of sensorimotor function, the reliability of the measure remains relatively low. Therefore, to improve the translation of afferent inhibition within and beyond the research lab, the reliability of the measure must be improved. Previous literature suggests that the focus of attention can modify the magnitude of afferent inhibition. As such, controlling the focus of attention may be one method to improve the reliability of afferent inhibition. In the present study, the magnitude and reliability of SAI and LAI was assessed under four conditions with varying attentional demands focused on the somatosensory input that evokes SAI and LAI circuits. Thirty individuals participated in four conditions; three conditions were identical in their physical parameters and varied only in the focus of directed attention (visual attend, tactile attend, non- directed attend) and one condition consisted of no external physical parameters (no stimulation). Reliability was measured by repeating conditions at three time points to assess intrasession and intersession reliability. Results indicate that the magnitude of SAI and LAI were not modulated by attention. However, the reliability of SAI demonstrated increased intrasession and intersession reliability compared to the no stimulation condition. The reliability of LAI was unaffected by the attention conditions. This research demonstrates the impact of attention/arousal on the reliability of afferent inhibition and has identified new parameters to inform the design of TMS research to improve reliability.

## Introduction

In humans, non-invasive brain stimulation (NIBS) has gained popularity due to its ability to modulate brain activity without the need for invasive procedures. Two popular methods of NIBS include transcranial magnetic stimulation (TMS) and transcranial electric stimulation (TES) [1]. When either method is delivered over the primary motor cortex, an increase in corticospinal excitability occurs that can be quantified via motor evoked potentials recorded using electromyography (EMG) [2]. TES yields the direct excitation of pyramidal tract axons within the white subcortical matter, eliciting a direct wave (D wave) [3]. In contrast, TMS transsynaptically activates pyramidal neurons, and elicits indirect waves (I waves) [3]. As the intensity of TMS increases, more I- waves are recruited which are a result of increased intracortical interneurons being activated [4].

When a single pulse of TMS is delivered to M1, upper and lower motor neurons in the corticospinal tract are depolarized, thus leading to a response in the target muscle [3]. As the intensity of TMS increases, the peak-to-peak amplitude of the MEP increases in a sigmoidal fashion [5] and reflects the recruitment of upper and lower motor neurons. TMS can be delivered as a single, dual, or repetitive pulse patterns [3, 6]. Single pulse patterns are typical used to quantify changes in corticospinal excitability [7] while repetitive and/or patterned TMS temporarily increases or decreases the excitability of cortical areas [7] and are used to aid in the understanding of cognitive processes and disorders [8–14].

When single pulse TMS is preceded by electrical stimulation to a peripheral nerve, a decrease in the MEP amplitude from the target area is observed, a phenomenon known as afferent inhibition. Afferent inhibition can be divided into two subtypes depending on the interstimulus interval (ISI) between the peripheral nerve stimulation and TMS: ISIs of ~20–25 ms are classified as short-latency afferent inhibition (SAI), whereas ISI of 200–1000 ms are classified as long-latency afferent inhibition (LAI) [15, 16]. SAI is thought to result from somatosensory afferent input, evoked by the peripheral nerve stimulation, that suppresses late I2 and I3 waves [16]. The mechanisms that underlie LAI remain unknown [16]. The strength of afferent inhibition correlates with the amplitude of the sensory afferent volley [17–19] such that greater inhibition is associated with a greater volume of sensory afference [20, 21]. There is speculation that SAI and LAI reflect cortical rather than spinal inhibitory mechanisms [15, 16, 22]. SAI and LAI have been acquired in multiple clinical populations including Parkinson’s disease [23–26], Alzheimer’s disease and dementia [27–29]. Alterations in SAI/LAI have been correlated with slower movements [30], motor disturbances [31] and cognition [32].

One challenge for research using SAI and LAI relates to reliability, which evaluates the consistency of a measure over time. Reliability assessments can be subdivided into two categories: absolute and relative reliability. Absolute reliability evaluates how a metric change over time in a stable individual, allowing for measurement error to be estimated [33, 34]. Absolute reliability is quantified with the standard error of measurement (SEM_eas_) and smallest detectable change (SDC) calculations [33]. The SEM_eas_ allows researchers to uncover how measures in a stable individual change over time, quantifying within-subject variability [33]. The SDC outlines the minimum amount of change needed to be observed for the change to be greater than expected due to random error [34]. In contrast, relative reliability refers to the ability of a metric to distinguish individuals from each other [34, 35]. Relative reliability can be assessed using the Intraclass Correlation Coefficient (ICC). ICCs reflect the ability of a test or measure to accurately differentiate between individuals [34]. The ICC is dependent on the sample, meaning a sample with a large amount of between subject variability will indicate high relative reliability of the measure, even if there is a large amount of error. This indicates that the ICC is context specific, and the heterogeneity of the sample collected is imperative to the calculation [34].

Tests of reliability in TMS evoked responses have become common. MEPs and resting motor threshold have demonstrated good test re-test reliability in contrast to measures of intracortical facilitation [36]. Researchers have also explored the reliability of afferent inhibition and concluded that SAI and LAI demonstrate poor to moderate reliability [37, 38]. The relative reliability of afferent inhibition measures across studies also seem to vary substantially, indicating that there may be methodological factors including coil placement, electrode placement [39] that are modulating reliability [40].

Physiological processes such as attention and arousal can affect neurophysiological measures [8, 41, 42] including SAI and LAI [43–46]. For example, afferent inhibition increased when attention was directed to the stimulated vs non-stimulated hand [46]. Attentional load can also modulate the depth of afferent inhibition, such that SAI is decreased during periods of high attentional load but is increased when attentional load is decreased [44]. These findings suggest that attention modulates SAI magnitude and therefore it is plausible that reliabilty of afferent inhibition can also be modulated by the focus of attention. The literature highlights that the relationship between afferent inhibition and human behaviour is relatively unclear [43]. Research has shown that afferent inhibition could potentially be modulated with attention [45, 46]. These findings suggest that attention could potentially influence the process of sensorimotor integration creating more reliable SAI and LAI. How might attention modulate SAI? The literature suggest that attention will cause changes in cortex activation, which could cause an enhancement in the afferent volley going to the cortex. Studies demonstrate that attention to specific features of a sensory stimulus leads to activation in the cortical region responsible for processing that stimulus feature [47, 48]. Based on the literature, it is possible that attention directed towards sensory mechanisms involved in afferent inhibition could lead to greater inhibition.

The purpose of the present study was to investigate the effects of attention on the reliability of SAI and LAI. Specifically, it was hypothesized that focusing attention on the somatosensory input eliciting SAI and LAI would increase the reliability of these measures. The results from this study are intended to aid in the design of future TMS studies and the usage of SAI and LAI in basic and clinical neuroscience settings.

## Materials and methods

### Participants

30 right-handed, healthy participants (15 females; age = 21.50 ± 3.00 years) were recruited. This sample size is in line with previous studies which have explored attention and afferent inhibition [44–46] but also studies which have aimed to explore the reliability of afferent inhibition [37]. Participants attended three separate sessions all scheduled in the afternoon to control for the effects of diurnal cortisol levels which may influence TMS measures [49]. Session 1 and 2 were separated by 60 mins and session 1 and 3 were separated by 1–7 days. Participants passed a screening for TMS contraindications [50] and were identified as right-handed using a modified version of the Edinburgh handedness questionnaire which required them to indicate which hand they use for several common manual tasks [51]. The participants provided written consent before the onset of the experiment. This study was approved by the Hamilton Integrated Research Ethics Board (HiREB) and conformed to the declaration of Helsinki.

### Electromyography

Surface electrodes (9 mm Ag-Cl) were used to record activity from the abductor pollicis brevis (ABP) muscle of the right hand. In order to reduce signal noise, a dry ground was placed on the styloid process at the right wrist. EMG signals were magnified x1000 and bandpass filtered between 20 Hz-2.5 kHz (Intronix Technologies Corporation Model 2024F, Bolton, Canada). An analog-digital converter was used to digitize data at 5 kHz (Power1401; Cambridge Electronics Design, Cambridge, UK), prior to being analyzed through commercial software (Signal v7.01; Cambridge Electronics Design, Cambridge, UK).

### Electroencephalography

Electroencephalography (EEG) electrodes were used to acquire somatosensory-evoked potentials (SEPs) from the primary somatosensory cortex (S1 Data). Electrodes were placed at C3’ (2 cm posterior to C3) with signals referenced to Fpz (international 10–20 system). A ground electrode was placed on the right clavicle. A bar electrode with the anode positioned distally was used to stimulate the median nerve at the wrist. 500 stimuli (200 μs square wave pulses; 3 Hz) were delivered at the minimum intensity needed to observe a visual contraction in the right APB muscle using a constant current stimulator (Digitimer DS7AH, Hertfordshire, UK). Resultant signals were averaged over the 500 epochs to identify the latency of the N20 component of the SEP. The N20 latency+ 4 ms was used to elicit SAI [16, 37, 52, 53].

### Transcranial magnetic stimulation

TMS was performed using a Magstim 200^2^ stimulator (Magstim, Whitland, UK). A 50 mm figure-of-eight branding coil was positioned over the left M1 at the optimal location to evoke MEPs from the right APB muscle. The coil was oriented at a 45-degree angle to the sagittal plane to induce a posterior-to-anterior current. The hotspot of the right APB muscle was defined as the location on the left M1 that, when stimulated with TMS, consistently led to a large MEP in the muscle [54]. This point was found and registered using Brainsight Neuronavigation (Rogue Research, Montreal, Canada).

#### Resting motor threshold

Resting motor threshold (RMT) was defined as the stimulus intensity (%MSO) that evokes and MEP (i.e. peak-to-peak amplitude >50 μV) 50% of the time [54]. This value was determined using TMS_MTAT_2.0 freeware (http://clinicalresearcher.org/software.htm). The stimulus intensity was set to 37%MSO, and twenty TMS pulses were distributed over M1, specifically the APB hotspot, with the stimulus intensity being adjusted after each subsequent pulse as advised by the MTAT software based on the presence or absence of an MEP on the previous trial [55].

#### Afferent inhibition

**Fig 1:**
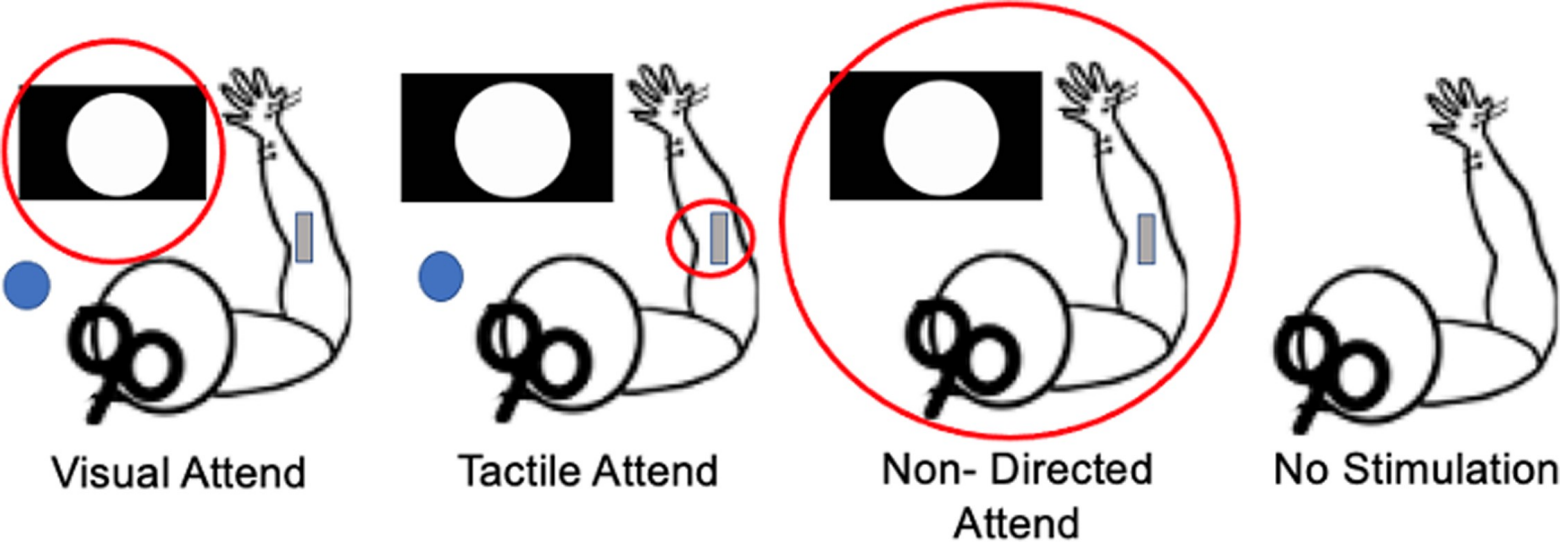
Experimental parameters. Participants were seated in front of a visual stimulus while receiving nerve stimulation and TMS. A) Tactile Attend B) Visual Attend C) Non-directed Attend D) No Stimulation. The red circles represent the experimental environment where the stimulus is present.

Afferent inhibition was acquired by delivering peripheral nerve stimulation directly prior to TMS. The intensity of TMS was adjusted to evoke a ~1 mV peak-to-peak amplitude MEP in the APB muscle [37, 44]. The right median nerve was stimulated using a constant current stimulator (Digitimer DS7AH, Hertfordshire, UK) at 200 μs square wave pulses, and at the intensity that corresponded to motor threshold of the APB muscle [20, 21, 37], which reflects the intensity at which maximum afferent inhibition is observed [4]. For SAI, the ISI between peripheral nerve stimulation and TMS was set to the latency of the N20 + 4ms [16, 17, 37, 52, 53] whereas for LAI the ISI was 200ms [2, 9, 17]. SAI and LAI were delivered at random within each condition separated by an inter-trial interval of approximately 6–8 seconds, therefore ensuring that participants could not predict the onset of afferent inhibition. Within each condition, 20 SAI MEP_condditioned_, 20 LAI MEP_conditioned_ and 20 MEP_unconditioned_ were evoked. The conditions are described in Fig 1.

### Experimental parameters

The experiment consisted of four conditions: *Visual Attend (VA)*, *Tactile Attend (TA)*, *Non-Directed Attend (NDA)*, *No Stimulation (NS)*. Fig 1 outlines the parameters of each condition.

#### Visual stimulation used in visual, tactile and non-directed attend conditions

The visual stimulus for this experiment consisted of a white circle appearing on the black screen, coded in MATLAB software (MATLAB and Statistics Toolbox Release 2012b, The MathWorks, Inc., Natick, Massachusetts, United States). Throughout the condition the intensity of brightness fluctuated, and participants were probed to respond to this fluctuation with a button press (Adapted/Modified from Hsiao, Lane, and Fitzgerald [56]). The number of intensity fluctuations for each condition was set to twenty changes and each participants onset of change was randomized for each condition.

#### Tactile stimulation used in visual, tactile and non-directed attend conditions

Individuals received several electrical stimuli to the right median nerve, at a location proximal to the median nerve stimulus delivered for TMS. The intensity of the tactile stimulation was set to sensory threshold, which reflects the intensity required for the individual to detect the stimuli [37]. To determine sensory threshold, a suprathreshold intensity was applied and individuals were asked to report whether or not they could perceive the stimulus. The stimulus intensity was then modulated in increments of 0.1mA to determine the minimal intensity required to identify the stimulus [20, 21, 37].

Duration of the pulse was approximately 200 μs square wave pulses. The nerve stimulus varied as a single pulse, double pulse, or triple pulse (adapted and modified from Kobt [46]). Depending on the condition, the participant was asked to respond with a button press when a triple-pulsed electrical stimuli is received. For *Visual Attend (VA)*, *Tactile Attend (TA)*, and *Non- Directed Attend (NDA)* 20 triple pulse nerve stimuli were delivered.

### Data analyses

Peak-to-peak EMG trials with activity exceeding 50 μV in a 100 ms window proceeding the TMS artefact were removed [35, 37]. The magnitude of afferent inhibition present was expressed as a ratio of the conditioned MEP amplitude to the unconditioned MEP amplitude (*SAI or LAI* = *MEP*_*CONDITIONED*_/*MEP*_*UNCONDITIONED*_ * 100%). Repeated Measures ANOVA with factor STATE was conducted to determine whether or not the *MEP*_*conditioned*_ is significant supressed relative to *MEP*_*unconditioned*_.

To assess the effects of attention on the magnitude of SAI and LAI, one-way Repeated Measures ANOVAs with factor CONDITION were completed using data collected at timepoint 1. Furthermore, the normality was determined through the Shapiro-Wilk test. If data was considered non-parametric, a Friedman’s ANOVA was conducted with a corresponding Wilcoxon Signed Rank post-hoc analysis. Post-Hoc tests were conducted using Tukey’s Honest Significant Difference (HSD), which examined all relevant pairwise comparisons between groups. Significance was set to α< 0.05. The accuracy data acquired in the VA and TA conditions was analysed using a Wilcoxon Signed Rank test.

To assess the effects of attention on the reliability of SAI and LAI, normality was assessed using Shapiro-Wilks tests, and heteroscedasticity was assessed using Bland-Altman plots. Violations of normality normally require a transformation on the dataset, but due to the reliability analysis that must take place, transformations were not implemented as it would cause a change in the ratio scale [57–59]. Bland-Altman plots were created comparing the respective variables at T1-T2, and T1-T3. Outliers were identified and removed using Grubb’s Test. Paired t-tests were used to compare each condition for both intrasession and intersession time points to discover if systematic error was present. Significance was set to alpha < 0.05. Relative reliability was quantified by averages of MEP amplitudes for all subsequent trials. ICCs were calculated for SAI and LAI using all data points within the condition. The data was obtained by one experimenter and hence, the ICC (2,k) model was implemented [34]. ICCs were evaluated using recommended guidelines where ICC with 95% CI above 0.9 is Excellent; 0.75 < ICC < 0.9 is High; 0.5 < ICC < 0.75 is Moderate; and ICC < 0.5 is considered Low [34, 60, 61]. Absolute reliability was determined for each condition using the SEM_eas_ values ($SEM_{eas}=\sqrt{MeanSquared_{error}}$) [33], which were then converted to represent %SEM_eas_ values ($\%SEM_{eas}=\frac{SEM_{eas}}{mean}*100\%$) [33]. %SEMeas < 10% was used as a cut off to indicate low measurement error [35]. Furthermore, the SEM_eas_ was used to determine the SDC_individual_ (*SDC*_individual_ = *SEMeas* × 1.96 × √2) and SDC_group_ ($SDC_{group}=\frac{SDC_{indiv}}{\sqrt{n}}$), where *n* is the sample size [34, 37].

## Results

All participants underwent the experimental manipulation with no adverse effects. Table 1 displays the group-averaged dependent measures across all three time points of acquisition. These data reveal no significant differences in the current delivered via nerve stimulation at motor threshold (Friedman’s ANOVA, χ^2^(2) = 3.045, p = 0.218), tactile stimulation delivered at sensory threshold (Friedman’s ANOVA, χ^2^(2) = 1.19, p = 0.551), SAI N20+4 latency (Wilcoxon signed-ranks; Z = -0.378, p = 0.705), RMT (Wilcoxon signed-ranks, Z = -0.332, p = 0.74) and %MSO to evoke a peak-to-peak 1mV MEP (Friedman’s ANOVA, χ^2^(2) = 2.53, p = 0.282).

**Table 1:** Group averaged measures (with standard deviations). SAI acquired with an ISI of N20+4ms, evoked by median nerve stimulation.

	T1(n = 30)	T2(n = 30)	T3(n = 30)
**Nerve Stimulation Motor Threshold (mA)**	5.98 ± 1.51	6.24 ±1.84	6.23 ±1.84
**Tactile Stimulation Sensory Threshold (mA)**	0.42 ±0.13	0.43 ±0.14	0.43 ±0.15
**SAI N20+4 latency (ms)**	21.9 ±0.92	-	21.9 ±0.87
**TMS Resting Motor Threshold (%MSO)**	46.57 ±6.16	-	46.37 ±6.50
**1mV as a %RMT**	125.73± 11.25	128.02 ±12.64	125.34 ± 10.46

%RMT: resting motor threshold, SAI: short latency afferent inhibition, %MSO = maximum stimulator output

Grubbs test indicated the removal of two outliers: an SAI data point from T2 and an LAI data point from T3. The assessment of the biological mechanism of attention utilized a sample of n = 30 to investigate the effects of attention on afferent inhibition. The reliability of SAI and LAI during various attention manipulations was addressed with n = 30, expect for the *VA* SAI T1-T2 and *TA—*LAI at T1- T3 which utilized a sample size of n = 29 for the following intersession analysis.

**Fig 2:**
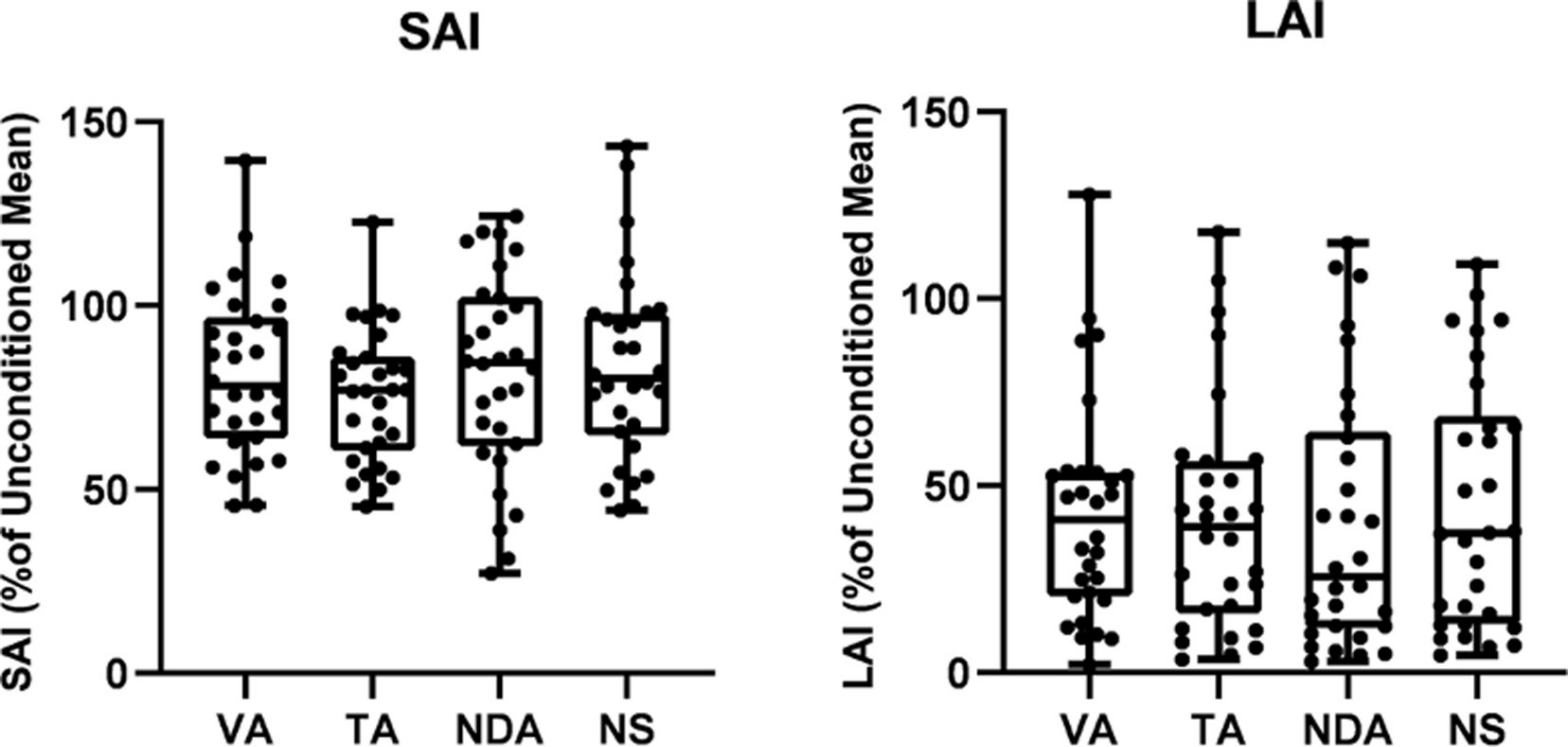
Biological effects of attention. A. Average SAI, expressed as a % of the unconditioned mean at. T1. Shown is the mean with whiskers spanning 1.5Χ the interquartile range. B. Average LAI, expressed as a % of the unconditioned mean. Shown is the mean with whiskers spanning 1.5Χ the interquartile range *LAI*: *long latency afferent inhibition*, *NDA*: *non-directed attend*, *NS*: *no stimulation*, *SAI*: *short latency afferent inhibition*, *TA*: *tactile attend*, *VA*: *visual attend*.

A Repeated measures ANOVA confirmed a main effect of STATE (F_(1,29)_ = 38.326, p<0.001, ηp2 = 0.970) such that MEP_conditioned_ is significantly supressed relative to MEP_unconditioned_ indicating that significant inhibition was observed in all conditions. Fig 2A plots the group-averaged mean with whiskers spanning 1.5 X interquartile range of SAI across all conditions. A One-way ANOVA revealed no effect of CONDITION (F _(3,87)_ = 1.082, p = 0.361, ηp2 = 0.036). These data indicate that the magnitude of SAI was unaltered by the focus of attention.

For LAI, a repeated measures ANOVA indicated a main effect of STATE (F_(1,29)_ = 102.90, p<0.001, ηp2 = 0.780) indicating significant suppression of the MEP in the presence of nerve stimulation. Friedman’s ANOVA was implemented to examine the effects of attention on LAI. The results shown in Fig 2B indicate no statistical difference in the magnitude of LAI across conditions (Friedman’s ANOVA, χ^2^(3) = 1.080, p = 0.782).

The performance data for *VA* and *TA* indicated that individuals had a higher % correct on the *TA* (mean rank = 15.84) when compared to *VA* (Wilcoxon signed-ranks; mean rank = 5.50), Z = -4.681, p<0.001).

The SAI dataset was normally distributed across all time points and conditions. In contrast, the LAI dataset was normally distributed for all LAI datapoints except at T3. Paired t-tests were completed for each condition at its corresponding timepoint, which indicated that no systematic error was present within comparisons of conditions at T1 vs T2 and T1 vs T3 for SAI and LAI (p>0.05). Homoscedasticity, determined via Bland Altman plots, was preserved for SAI at T1-T2 and T1-T3; however, for LAI, it was upheld at T1-T3 but was heteroscedastic for T1-T2 specifically for the *NS* condition, with an R^2^>0.1. Due to the heteroscedastic nature of LAI at T1-T2, usually a correction via log transformation can be implemented on the dataset; however, given that the transformations change the data to a ratio scale. the transformation was not performed, and the data was instead analyzed with an assumption of heteroscedasticity as done in previous studies [57–59].

### SAI

SAI at the intrasession level demonstrates poor reliability for the *NS* condition (ICC = 0.10) compared to the moderate reliability obtained in all attention manipulation conditions (*VA*, *TA* and *NDA*). For all conditions, the %SEM_eas_ demonstrates large amounts of measurement error (%SEM >10%) (Table 2). The SDC_individual_ indicates that a minimum change of 52, 45, 59 and 70% is needed to be considered physiological change at the individual level for the *VA*, *TA*, *NDA* and *NS*, respectively (Table 2). In summary, each condition requires a large physiological change to occur to be considered a real change at the level of an individual. Furthermore, in order for the SDC_Group_ to be <10%, at which it is feasible to see a real physiological change at the group level, a sample size of 24 is needed for *VA*, 19 is needed for *TA*, 32 is needed for *NDA* and 45 is needed for *NS*.

**Table 2:** Intrasession reliability statistics SAI. SEM_eas_, SDC_individual,_ SDC_group_ are expressed as a percentage of the unconditioned MEP. In contrast, %SEM_eas_ is expresses the SEM_eas_ as a percentage of the mean.

Intrasession SAI	ICC (95% CI)	SEM_eas_	SEM_eas_%	SDC_group_	SDC_Individual_
*VA*	0.45 (-0.161 to 0.741)	18.89%	24.01%	9.72%	52.36%
*TA*	0.56 (0.06 to 0.791)	16.27%	21.55%	8.24%	45.12%
*NDA*	0.44 (-0.186 to 0.732)	21.40%	26.97%	10.83%	59.31%
*NS*	0.101 (-0.88 to 0.57)	25.31%	31.62%	12.81%	70.15%

CI: confidence Interval, ICC: intraclass correlational coefficient, NDA: non-directed attend, NS: no stimulation, SDC: smallest detectable change, SEM_eas_: standard error of measurement, %SEM_eas_: relative SEM_eas._ TA: tactile attend, VA: visual attend.

SAI at the intersession level also demonstrates poor reliability for the *NS* condition (ICC = 0.25) compared to the moderate reliability obtained in all attention manipulation conditions (*VA*, *TA* and *NDA*). For all conditions, the % SEM_eas_ demonstrates large amounts of measurement error (%SEM_eas_ >10%) (refer to Table 3). The SDC_individual_ indicates that a minimum change of 65, 47, 61 and 64% is needed to be considered physiological change at an individual level for the *VA*, *TA*, *NDA* and *NS*, respectively (Table 3). This indicates each condition requires a large physiological change to occur to be considered a real change at the level of the individual. Furthermore, in order for the SDC_Group_ to be <10%, at which it is feasible to see a real physiological change at the group level to see a real physiological change at the group level, a sample size of 39 is needed for *VA*, 21 is needed for *TA*, 34 is needed for *NDA* and 37 is needed for *NS*.

**Table 3:** Intersession reliability statistics SAI. SEM_eas_, SDC_individual,_ SDC_group_ are expressed as a percentage of the unconditioned MEP. In contrast, %SEM_eas_ is expresses the SEM_eas_ as a percentage of the mean.

Intersession SAI	ICC (95% CI)	SEM_eas_	SEM_eas_%	SDC_group_	SDC_indivdual_
*VA*	0.42 (-0.22 to 0.72)	23.57%	27.94%	11.93%	65.34%
*TA*	0.41(-0.27 to 0.72)	16.98%	22.34%	8.59%	47.06%
*NDA*	0.54 (0.01 to 0.78)	22.18%	26.86%	11.22%	61.48%
*NS*	0.25 (-0.53 to 0.64)	23.06%	29.19%	11.67%	63.92%

CI: confidence Interval, ICC: intraclass correlational coefficient, NDA: non-directed attend, NS: no stimulation, SDC: smallest detectable change, SEM_eas_: standard error of measurement, %SEM_eas_: relative SEM_eas._ TA: tactile attend, VA: visual attend.

### LAI

As shown in Table 4, LAI at the intrasession level demonstrates high relative reliability (0.75 <ICC< 0.9) for all attention manipulation conditions (*VA*, *TA*, *NDA*) and moderate reliability (0.5<ICC<0.75) for the *NS* condition. For all conditions, the % SEM_eas_ demonstrates large amounts of measurement error (%SEM_eas_ >10%) (Table 4). The SDC_individual_ indicates that a minimum change of 41, 50, 47 and 59% is needed to be considered physiological change at an individual level for the *VA*, *TA*, *NDA* and *NS*, respectively (Table 4). In summary, each condition requires a large physiological change to occur to be considered a real change at the level of an individual. Furthermore, in order for the SDC_Group_ to be <10%, at which it is feasible to see a real physiological change at the group level, a sample size of 16 is needed for *VA*, 23 is needed for *TA*, 21 is needed for *NDA* and 32 is needed for *NS*.

**Table 4:** Intrasession reliability statistics LAI. SEM_eas_, SDC_individual,_ SDC_group_ are expressed as a percentage of the unconditioned MEP. In contrast, %SEM_eas_ is expresses the SEM_eas_ as a percentage of the mean.

Intrasession LAI	ICC (95% CI)	SEM_eas_	SEM_eas_ %	SDC_group_	SDC_individual_
*VA*	0.86 (0.71 to 0.93)	14.97%	32.97%	7.58%	41.52%
*TA*	0.82 (0.63 to 0.92)	18.15%	41.09%	9.19%	50.32%
*NDA*	0.85 (0.69 to 0.93)	17.15%	40.42%	8.68%	47.54%
*NS*	0.75 (0.47 to 0.89)	21.51%	47.13%	10.88%	59.64%

CI: confidence Interval, ICC: intraclass correlational coefficient, NDA: non-directed Attend, NS: no Stimulation, SDC: smallest detectable change, SEM_eas_: standard error of measurement, %SEM_eas_: relative SEM_eas._ TA: tactile attend, VA: visual attend.

Similarly, LAI at the intersession level demonstrates high relative reliability (0.75 <ICC< 0.9) for all conditions (*VA*, *TA*, *NDA*) and moderate reliability (0.5<ICC<0.75) for the *NS* condition as well. For all conditions, the %SEM_eas_ demonstrate large amounts of measurement error (%SEM_eas_ >10%) (Table 5). The SDC_individual_ indicates that a minimum change of 55, 48, 48 and 54% is needed to be considered physiological change at an individual level for the *VA*, *TA*, *NDA* and *NS*, respectively (Table 4). In conclusion, each condition requires a large physiological change to occur to be considered a real change at the level of an individual. Furthermore, in order for the SDC_Group_ to be <10%, at which it is feasible to see a real physiological change at the group level, a sample size of 29 is needed for *VA*, 21 is needed for *TA*, 21 is needed for *NDA*. and 28 is needed for *NS*.

**Table 5:** Intersession reliability statistics LAI. SEM_eas_, SDC_individual,_ SDC_group_ are expressed as a percentage of the unconditioned MEP. In contrast, %SEM_eas_ is expresses the SEM_eas_ as a percentage of the mean.

Intersession LAI	ICC (95% CI)	SEMeas	SEMeas%	SDC_group_	SDC_individual_
*VA*	0.76 (0.49 to 0.88)	20.00%	44.02%	10.12%	55.45%
*TA*	0.80 (0.56 to 0.90)	17.44%	43.64%	8.98%	48.34%
*NDA*	0.86 (0.70 to 0.93)	17.49%	41.55%	8.85%	48.48%
*NS*	0.73 (0.43 to 0.87)	19.68%	47.00%	9.96%	54.55%

CI: confidence Interval, ICC: intraclass correlational coefficient, NDA: non-directed Attend, NS: no Stimulation, SDC: smallest detectable change, SEM_eas_: standard error of measurement, %SEM_eas_: relative SEM_eas._ TA: tactile attend, VA: visual attend.

## Discussion

The purpose of the present study was to investigate the influence of attention on the reliability of SAI and LAI. Overall, LAI had higher levels of relative reliability compared to SAI across all four conditions. Further, for SAI, relative reliability was higher for conditions where attention was manipulated compared to the no stimulation condition. Last, in contrast to previous findings, attention did not modulate the depth of SAI and LAI. These findings and potential mechanisms are discussed.

For SAI, all attention conditions yielded similar improvements in reliability, even the condition in which attention was not directed specifically at the visual or tactile stimulation. Specifically, in the no stimulation condition, which yielded low reliability, there were no visual elements included in the participants environment (i.e. no computer interface to view, no tactile stimulation to direct attention towards). Therefore, all attention conditions yielded improvements in reliability, and these effects may be due to attention towards external features in the experimental environment. These data appear to indicate that having a stimulating environment may in of itself improve the reliability of afferent inhibition.

Arousal may provide one explanation to explain why environmental stimuli may improve the reliability of SAI. Arousal appears to affect the magnitude of SAI such that lorazepam (GABA_A_ agonist) that causes sedation also reduces SAI/LAI [62–64]. In contrast, baclofen (GABA_B_ agonist) which does not cause sedation, does not change SAI/LAI [63]. It is possible that when individuals have minimal external stimuli, such as in the *NS* condition, motivation and arousal states decrease, which may contribute to the reduced SAI reliability during the *NS* condition in this experiment. Conversely, in the attention tasks, the greater arousal created by the visual environment yields improvements in reliability.

It is also likely that attention to the somatosensory stimuli led to the improved reliability of SAI. Attention to somatosensory stimuli may alter SAI via increased processing of the afferent volley at any of the synapses along the ascending axis. For example, in non-human primates, relevant stimuli increase the firing rate of dorsal horn [65], thalamic [66, 67], and S1 Data [68]. It is possible that orienting attention would enhance sensory processing in this pathway, thereby yielding a stronger sensory signal. The greater sensory afferent projection would ultimately act on inhibitory interneurons in the motor cortex enhancing afferent inhibition [47, 69]. Although these mechanisms may contribute to the improved reliability of SAI, they do not explain the improved reliability observed in the ‘non-directed attention’ condition which may be due to arousal.

The prefrontal cortex, which contributes to the phenomena of sensory gating, may modulate SAI. Sensory gating is the ability to filter irrelevant from relevant sensory information [70]. Enhanced sensory gating has been linked with reduced distractibility and faster reaction times in the continuous performance of sustained attention tasks [71, 72]. Studies demonstrate that when attention is directed towards a relevant somatosensory stimulus, enhanced sensory gating of neural somatosensory responses, related to ‘bottom up’ stimulus processing, is observed [73]. Wiesman *et al* [70] examined the relationship between directed attention and somatosensory gating. Participants were instructed to direct their attention either towards or away from a somato-visual -paired pulse oddball paradigm [70]. Results indicated an increase in alpha coherence between the prefrontal and somatosensory cortices when attention was directed towards somatosensory stimulation [70]. Activation of the prefrontal cortex has also been linked to attention directed towards tactile stimuli [74] while damage is associated with deficits in sensory gating and sustained attention [75]. Therefore, in the present study, it is possible that attention to somatosensory stimulation could be modulated by projections from the prefrontal cortex to primary somatosensory cortex (S1 Data).

The reliability of LAI was not heavily influenced by attention manipulations. The results of the reliability assessments for the intersession measures were similar to that of previous studies [37]. While much of the mechanism underlying LAI is currently unknown, studies have demonstrated that GABA_A_ agonists lead to significant reductions in LAI [52]. However, unlike SAI, no work to date has investigated whether a connection between LAI and the cholinergic system exists. In this study, the relationship between attention and LAI is not apparent. Given that attention is governed by the cholinergic system [30, 60, 61, 76], this effect may indicate that LAI is not cholinergic in nature, which has also been suggested by pharmacological studies [63].

This study did not demonstrate attention-related effects on the magnitude of SAI/LAI as shown elsewhere [44–46]. Suzuki et al., [45] evaluated the effect of working memory demands on sensory motor function. Participants were presented with a low working memory load (two digits) or a high working memory load (six digits), followed by a two second delay. Participants then had to indicate whether a presented number was a part of the previous set. SAI was reduced during the high working memory load condition compared to the low load condition, suggesting that working memory modulates the SAI pathway [45]. The reduction in SAI may be due to the suppression of sensory afferent input, which is irrelevant to the numeric working memory task. Importantly, working memory was not required in the attention tasks tested herein, and this may have led to the discrepant results. Further, Mirdamadi et al., [44] measured SAI during the performance of high versus low visual attention demand tasks [44]. Participants were provided with an array of crosses which varied in both colour and orientation. In the low demand condition participants were asked to count the number of red crosses regardless of orientation [44]. In the high demand task participants were asked to identify the number of upright yellow or inverted green crosses [44]. SAI was reduced during periods of high visual attention demands in comparison to periods of low visual attention demands [44]. Similarly, the present work did not employ a working memory component, and this may have yielded the differing outcome.

### Limitations and future directions

The present study was limited by sample demographics. The participants in this study consist of young adults only. Hence, future work should aim to address these questions in aging and special populations such as those with Alzheimer’s disease [77, 78], Mild cognitive impairment [79] and Parkinson’s disease [80–83]. In addition, the impact of external stimuli demonstrated an effect on the reliability of SAI. In the presence of an enriched external environment the reliability improved, but the exact features within the external environment which caused this change remains unknown. Future research should look to deconstruct the external environment to determine which features are responsible for the differences in reliability of SAI observed.

## Conclusion

This study investigated the effects of attention on the reliability of SAI and LAI. At present, it appears that the reliability of SAI is improved by arousal created by the experiment environment and/or attention demands. SAI is more reliable when recorded within the context of a stimulating experimental environment.

## Supporting information

S1 Data (XLSX)
